# The Role of Dopamine D3 Receptors, Dysbindin, and Their Functional Interaction in the Expression of Key Genes for Neuroplasticity and Neuroinflammation in the Mouse Brain

**DOI:** 10.3390/ijms24108699

**Published:** 2023-05-12

**Authors:** Veronica Rivi, Cristina Benatti, Joan M. C. Blom, Luca Pani, Nicoletta Brunello, Filippo Drago, Francesco Papaleo, Filippo Caraci, Federica Geraci, Sebastiano Alfio Torrisi, Gian Marco Leggio, Fabio Tascedda

**Affiliations:** 1 Department of Biomedical, Metabolic and Neural Sciences, University of Modena and Reggio Emilia, 41125 Modena, Italy; veronica.rivi@unimore.it (V.R.); cristina.benatti@unimore.it (C.B.); joan.blom@unimore.it (J.M.C.B.); luca.pani@unimore.it (L.P.); 2 Centre of Neuroscience and Neurotechnology, University of Modena and Reggio Emilia, 41125 Modena, Italy; 3 Department of Psychiatry and Behavioral Sciences, University of Miami, Miami, FL 33136, USA; 4 Department of Life Sciences, University of Modena and Reggio Emilia, 41125 Modena, Italy; nicoletta.brunello@unimore.it; 5 Department of Biomedical and Biotechnological Sciences, University of Catania, 95123 Catania, Italy; filippo.drago@unict.it (F.D.); filippo.caraci@unict.it (F.C.); federica.geraci@unict.it (F.G.); sebastiano.torrisi@unict.it (S.A.T.); 6 Department of Neuroscience and Brain Technologies, Italian Institute of Technology, 16132 Genova, Italy; francesco.papaleo@iit.it

**Keywords:** schizophrenia, BDNF, dopamine, glutamate, inflammation

## Abstract

Cognitive impairment in schizophrenia remains a clinically and pharmacologically unsolved challenge. Clinical and preclinical studies have revealed that the concomitant reduction in dysbindin (DYS) and dopamine receptor D3 functionality improves cognitive functions. However, the molecular machinery underlying this epistatic interaction has not yet been fully elucidated. The glutamate NMDA receptors and the neurotrophin BDNF, with their established role in promoting neuroplasticity, may be involved in the complex network regulated by the D3/DYS interaction. Furthermore, as inflammation is involved in the etiopathogenesis of several psychiatric diseases, including schizophrenia, the D3/DYS interaction may affect the expression levels of pro−inflammatory cytokines. Thus, by employing mutant mice bearing selective heterozygosis for D3 and/or DYS, we provide new insights into the functional interactions (single and synergic) between these schizophrenia susceptibility genes and the expression levels of key genes for neuroplasticity and neuroinflammation in three key brain areas for schizophrenia: the prefrontal cortex, striatum, and hippocampus. In the hippocampus, the epistatic interaction between D3 and DYS reversed to the wild-type level the downregulated mRNA levels of GRIN1 and GRIN2A were observed in DYS +/− and D3 +/− mice. In all the areas investigated, double mutant mice had higher BDNF levels compared to their single heterozygote counterparts, whereas D3 hypofunction resulted in higher pro−inflammatory cytokines. These results may help to clarify the genetic mechanisms and functional interactions involved in the etiology and development of schizophrenia.

## 1. Introduction

Schizophrenia is an idiopathic psychiatric disorder occurring in 0.5–1% of the general population [1,2]. Its symptoms include hallucinations, disorganized speech, abnormal motor behavior, and cognitive dysfunctions [3,4,5,6,7].

Despite decades of research, the etiology and pathological mechanisms of schizophrenia have not been fully elucidated yet [8,9,10]. This is in part due to the genetic and clinical heterogeneity of the disease [11,12,13,14]. Furthermore, efficient treatment of this disabling psychiatric disorder is not yet available, leaving huge unmet medical needs for patients [15,16,17].

In this context, Dystrobrevin Binding Protein 1 (DTBP1) gene, which encodes for Dysbindin (DYS), and the dopamine D2−like receptors (D2R and D3R) represent leading candidate susceptibility genes for schizophrenia [18,19,20,21].

In particular, a decreased D1/D2 activation ratio due to excessive D2 signaling may induce alterations of cortical network excitability, which in turn results in positive symptoms (i.e., delusions and hallucinations), negative symptoms, and cognitive impairments [22]. DYS is localized at both pre−and post−synaptic sites in the dorsolateral prefrontal cortex (PFC) and hippocampus (HIPP), where it regulates neurotransmitter release, receptors signaling, and intracellular protein trafficking involving lysosomes and related organelles [23].

Both D2−like dopaminergic receptors and the subunit 2A of the N−methyl−D−aspartate receptor (NMDA) glutamatergic receptors (GRIN2A) are trafficked after internalization via lysosomal−mediated degradation, suggesting that a reduction in DYS levels may selectively alter the dopaminergic and glutamatergic pathways, which, in turn, play a crucial role in the pathobiology of schizophrenia [24,25,26,27,28,29].

In particular, genetic disruption of DYS has been proven to alter the intracellular trafficking of D2−like receptors (and not the D1 ones), resulting in the increased insertion of D2 receptors on the neuronal surface [29]. Consistent with this observation, preclinical and clinical studies have demonstrated that genetic variations in DYS affect schizophrenia−relevant behavioral phenotypes and cognition through dopamine/D2−like mechanisms [30].

Therefore, given the strong connection between DYS function and D2−like receptors in the pathogenesis of schizophrenia, genetic variations that alter DYS functioning might predict important differences not only in the psychiatric phenotypes but also in the dosing and efficiency of drugs targeting D2 receptors, including several antipsychotic drugs [31].

On the other hand, Leggio et al. (2021) recently demonstrated that a concomitant reduction in D3R and DYS functionality correlates with the improvement of executive and working memory abilities in both rodents and patients with schizophrenia [32].

Therefore, as cognitive deficits in schizophrenia are widely considered to be core symptoms of this disease for which no adequate treatment strategy is available, an enhanced understanding of the biological functions and molecular pathways mediated by the DYS/D3 interaction is necessary and urgent [33].

In this study, by employing mutant mice bearing selective heterozygosis for D3 and/or DYS, we aimed to provide new insights into the functional interactions (single and synergic) between these schizophrenia susceptibility genes and neuroplasticity−related genes in brain areas implicated in both cognitive functions and negative symptoms of schizophrenia (i.e., prefrontal cortex, striatum, and hippocampus).

In particular, we focused our attention on two subtypes of the glutamate receptor—NMDA (1, 2A, and 2B) and the neurotrophin BDNF, which play a critical role in mediating cognitive functions that are impaired in schizophrenia [34,35,36,37].

Furthermore, in the same experimental conditions, we measured the expression levels of the main pro−inflammatory cytokines regulated by NF−kB (i.e., IL6, TNFα, and IL1β), given their proven role in the etiopathogenesis of several psychiatric diseases, including schizophrenia [38]. 

In fact, growing evidence has shown dopamine as a key regulator of inflammation [39], suggesting that alterations in dopamine levels associated with schizophrenia might affect inflammatory response and consequently some behavioral functions, including reference memory, learning, social behavior, and stress resilience [40,41,42,43,44,45]. Thus, we investigated the potential role (single and synergic) of DYS and D3 receptors and their functional interaction in regulating neuroplasticity genes and pro−inflammatory cascades.

## 2. Results

### 2.1. In the Striatum, D3 Hypofunctioning Upregulates DYS mRNA Levels, and DYS Hypofunctioning Upregulates D3 mRNA Levels, but These Upregulations Are Reversed to the WT Levels in Mice Bearing the Double Heterozygosis

**Figure 1:**
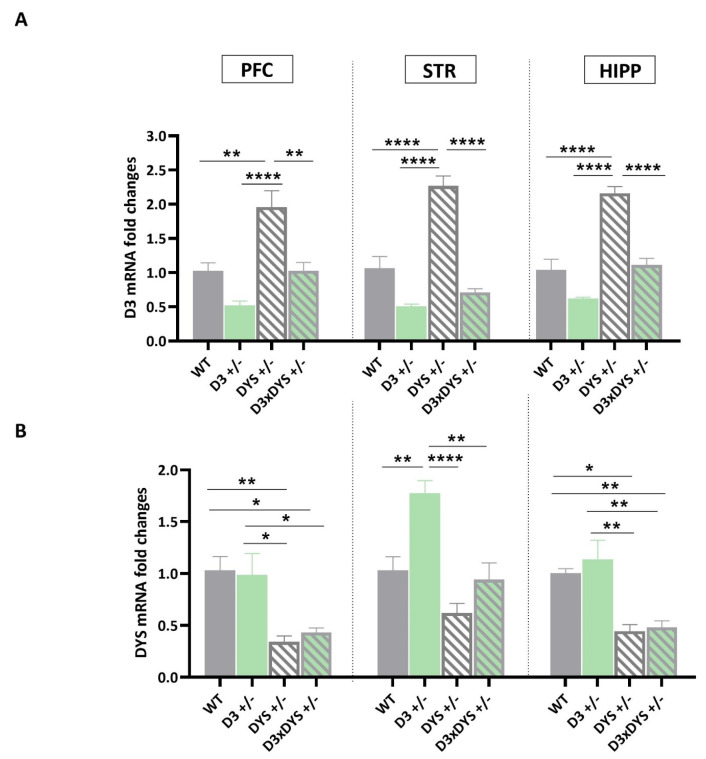
Transcriptional levels of dopamine D3 receptors (**A**) and Dysbindin (DYS) (**B**) in the prefrontal cortex (PFC), striatum (STR), and hippocampus (HIPP) of WT (D3×DYS +/+), D3 single heterozygous (D3 +/−), DYS single heterozygous (DYS +/−), and double D3 and DYS heterozygous (D3×DYS +/−) mice. Data are represented as means ± SEM and were analyzed with One−way ANOVA followed by Tukey’s HSD (* *p* < 0.05, ** *p* < 0.01, and **** *p* < 0.0001).

Consistent with previous studies [32], we found a significant upregulation of D3 mRNA levels (Figure 1A) in the PFC and striatum of DYS +/− mice (PFC: F3, 16 = 16.09, *p* < 0.0001; STR: F3, 16 = 46.06, *p* < 0.0001) compared to the other genotypes (PFC: WT vs. DYS +/−: *p* = 0.002; D3 +/− vs. DYS +/−: *p* < 0.0001; DYS +/− vs. D3×DYS +/−: *p* = 0.002; Striatum: WT vs. DYS +/−: *p* < 0.0001; D3 +/− vs. DYS +/−: *p* <0.0001; DYS +/− vs. D3×DYS +/−: *p* < 0.0001, Tuckey’s post hoc).

Furthermore, in this study, we observed that the same upregulation of the D3 mRNA levels occurred also in the HIPP (F3, 16 = 40.05, *p* < 0.0001; WT vs. DYS +/−: *p* < 0.0001; D3 +/− vs. DYS +/−: *p* < 0.0001; DYS +/− vs. D3×DYS +/−: *p* < 0.0001, Tuckey’s post hoc).

In all the areas, the D3 mRNA levels were reversed to the WT levels in double heterozygous mice (WT vs. D3×DYS +/−, PFC: *p* > 0.999; STR: *p* = 0.18; HIPP: *p* = 0.96, Tuckey’s post hoc). In contrast, single mutant−induced D3 hypofunctioning resulted in a significant upregulation of DYS only in the STR (F3, 16 = 14.55, *p* < 0.0001; Tuckey’s post hoc: D3 +/− vs. WT: *p* = 0.004; D3 +/− vs. DYS +/−: *p* < 0.0001; D3 +/− vs. D3×DYS +/−: *p* = 0.0015), without affecting its expression in the HIPP and PFC. This upregulation was reversed to the WT level in D3×DYS +/− mice (*p* = 0.96) (Figure 1B).

Unlike in the striatum, in both the PFC (F3, 16 = 8.03, *p* = 0.017) and HIPP (F3, 16 = 11.67, *p* = 0.0003), the mRNA levels of DYS were significantly downregulated in DYS +/− and D3×DYS +/− mice (PFC: DYS +/− vs. WT: *p* = 0.008; DYS +/− vs. D3 +/−: *p* = 0.012; D3×DYS +/− vs. WT: *p* = 0.02; D3×DYS +/− vs. D3 +/−: *p* = 0.032; HIPP: DYS +/− vs. WT: *p* = 0.007; DYS +/− vs. D3 +/−: *p* = 0.0012; D3×DYS +/− vs. WT: *p* = 0.013; D3×DYS +/− vs. D3 +/−: *p* = 0.002, Tuckey’s post hoc).

### 2.2. The Epistatic Interaction between D3 and DYS Reverses to the WT Levels the GRIN1 and GRIN2A Downregulation Observed in DYS +/− and D3 +/− Mice

**Figure 2:**
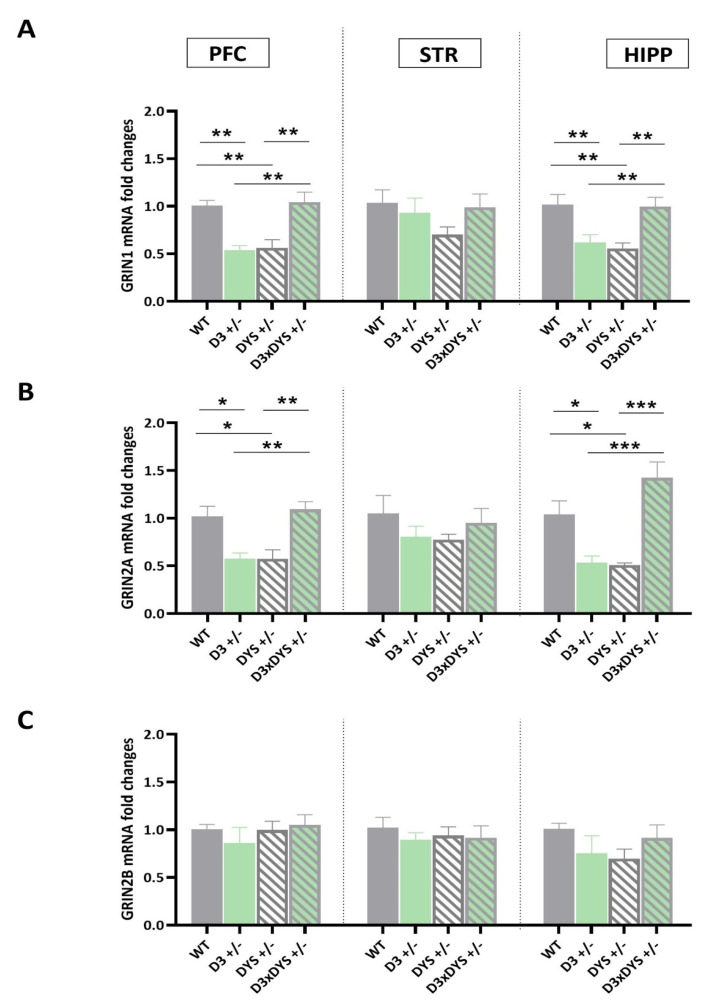
Transcriptional levels of GRIN1 (**A**), GRIN2A (**B**), and GRIN2B (**C**) in the prefrontal cortex (PFC), striatum (STR), and hippocampus (HIPP) of WT (D3×DYS +/+), D3 single heterozygous (D3 +/−), DYS single heterozygous (DYS +/−), and double D3 and DYS heterozygous (D3×DYS +/−) mice. Data are represented as means ± SEM and were analyzed with One−way ANOVA followed by Tukey’s HSD (* *p* < 0.05, ** *p* < 0.01, *** *p* < 0.001).

Next, we focused our attention on key genes for memory formation and cognitive functions: the subunits 1, 2A, and 2B of the NMDA ionotropic glutamatergic receptors (GRIN) (Figure 2).

The expression levels of GRIN1 were significantly downregulated in the PFC and HIPP (PFC: F 3,16 = 12.67, *p* = 0.0002; HIPP: F 3,16 = 7.98, *p* = 0.002) of D3 +/− and DYS +/− mice with respect to both the WT controls (PFC: DYS +/− vs. WT: *p* = 0.003; D3 +/− vs. WT: *p* = 0.0045; HIPP: DYS +/− vs. WT: *p* = 0.022; DYS +/− vs. WT: *p* = 0.008, Tuckey’s post hoc) and the double heterozygous counterparts (PFC: DYS +/− vs. D3×DYS +/−: *p* = 0.0014; D3 +/− vs. D3×DYS +/−: *p* = 0.0022; HIPP: DYS +/− vs. D3×DYS +/−: *p* = 0.03; DYS +/− vs. D3×DYS +/−: *p* = 0.011, Tuckey’s post hoc) (Figure 2A).

No difference in GRIN1 mRNA levels was reported between WT and D3×DYS +/− double mutant mice in both PFC and HIPP (PFC: *p* = 0.003; HIPP: *p* = 0.98, Tuckey’s post hoc).

Similar results were found for GRIN2A expression levels in both PFC and HIPP (PFC: F 3,16 = 10.41, *p* = 0.0005; HIPP: F 3,16 = 14.48, *p* < 0.0001).

In particular, Tuckey’s post hoc analysis showed that D3 or DYS and hypofunctioning were associated with a significant downregulation of the mRNA levels of this target compared to the WT (PFC: DYS +/− vs. WT: *p* = 0.01; D3 +/− vs. WT: *p* = 0.011; HIPP: DYS +/− vs. WT: *p* = 0.022; DYS +/− vs. WT: *p* = 0.03, Tuckey’s post hoc) and the double heterozygous ones (PFC: DYS +/− vs. D3×DYS +/−: *p* = 0.0032; D3 +/− vs. D3×DYS +/−: *p* = 0.0033; HIPP: DYS +/− vs. D3×DYS +/−: *p* = 0.0003; DYS +/− vs. D3×DYS +/−: *p* = 0.0002) (Figure 2B).

No significant differences were found in GRIN2A mRNA levels between WT and D3×DYS +/− mice in both PFC and HIPP (PFC: *p* = 0.93; HIPP: *p* = 0.98; DYS +/− vs. WT: *p* = 0.13, Tuckey’s post hoc). The striatal expression levels of GRIN1 and GRIN2A were not significantly different among the genotypes (F 3,16 = 1.38, *p* = 0.81; F 3,16 = 1.38; *p* = 0.93, *p* = 0.45, respectively).

Finally, GRIN2B mRNA levels were unchanged by alterations in either the DYS and D3 genotypes individually or interactively in all the areas examined (PFC: F 3,16 = 0.29, *p* = 0.82; STR: F 3,16 = 0.54, *p* = 0.66; HIPP: F 3,16 = 1.21, *p* = 0.33) (Figure 2C).

### 2.3. In All the Areas Investigated, Double Mutant Mice Have Higher BDNF mRNA Levels than Their Single Heterozygous Counterparts

**Figure 3:**
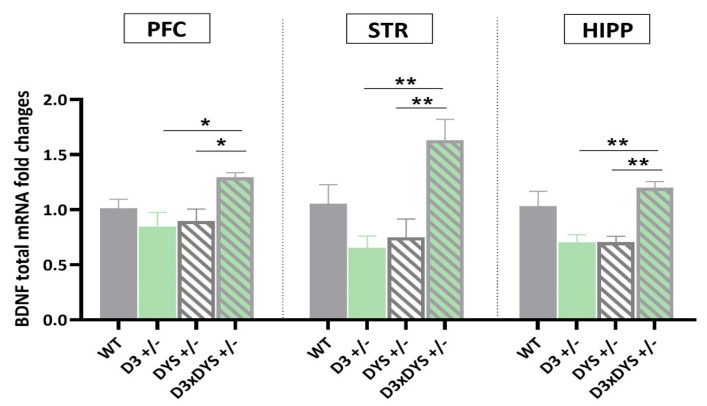
Transcriptional levels of BDNF in the prefrontal cortex (PFC), striatum (STR), and hippocampus (HIPP) of WT (D3×DYS +/+), D3 single heterozygous (D3 +/−), DYS single heterozygous (DYS +/−), and double D3 and DYS heterozygous (D3×DYS +/−) mice. Data are represented as means ± SEM and were analyzed with One−way ANOVA followed by Tukey’s HSD (* *p* < 0.05; ** *p* < 0.01).

Interestingly, one−way ANOVA revealed significant differences in BDNF mRNA levels between the genotypes (PFC: F 3,16 = 4.31, *p* = 0.02; STR: F 3,16 = 7.30, *p* = 0.003; HIPP: F 3,16 = 8.57, *p* = 0.0013) (Figure 3). In particular, in all the investigated areas, D3×DYS +/− double mutant mice showed an upregulation of BDNF mRNA levels compared to their single heterozygous counterparts (PFC: D3×DYS +/− vs. D3 +/−: *p* = 0.022; D3×DYS +/− vs. DYS +/−: *p* = 0.045; STR: D3×DYS +/− vs. D3 +/−: *p* = 0.003; D3×DYS +/− vs. DYS +/−: *p* = 0.007; HIPP: D3×DYS +/− vs. D3 +/−: *p* = 0.003; D3×DYS +/− vs. DYS +/−: *p* = 0.004, Tuckey’s post hoc).

### 2.4. D3 Hypofunction Results in Higher Pro−Inflammatory Cytokine Levels in the PFC and Striatum

**Figure 4:**
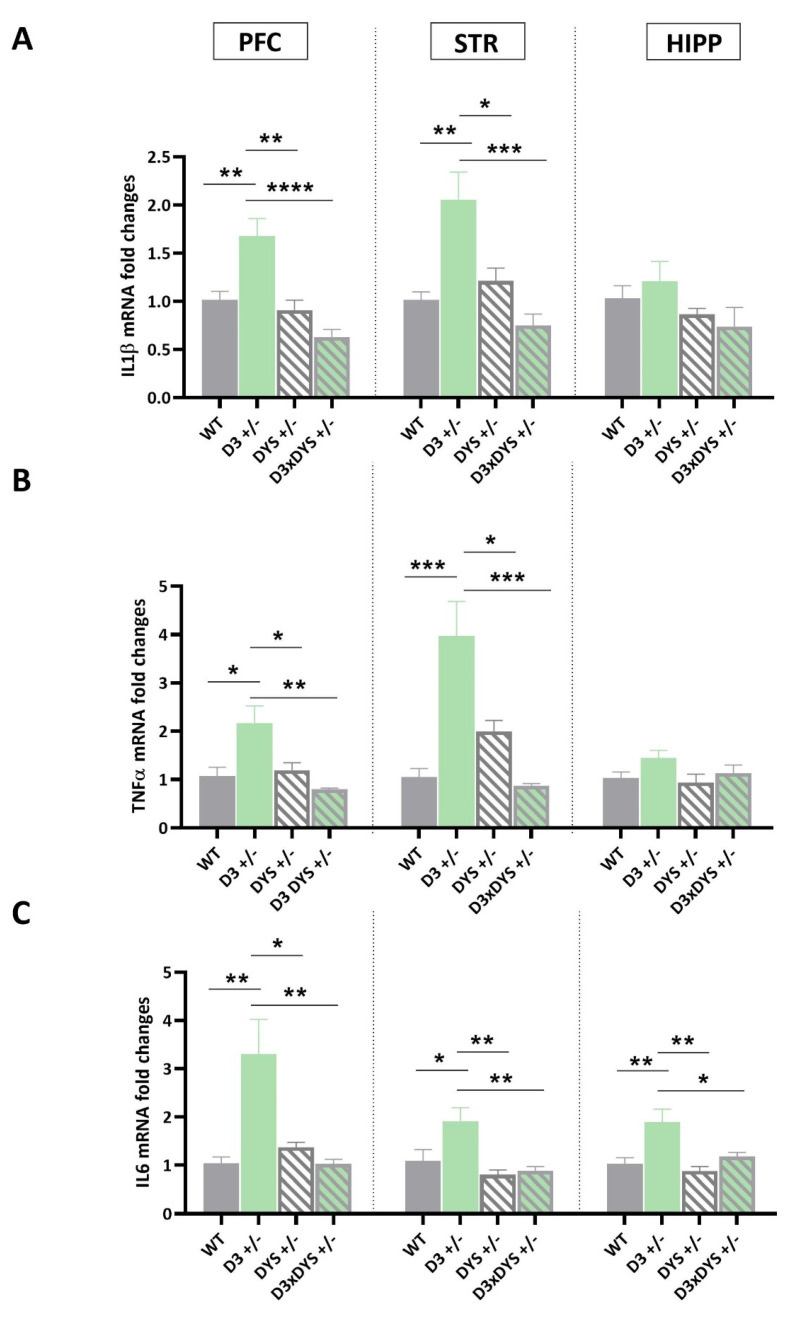
Transcriptional levels of IL1β (**A**), TNFα (**B**), and IL6 (**C**) in the prefrontal cortex (PFC), striatum (STR), and hippocampus (HIPP) of WT (D3×DYS +/+), D3 single heterozygous (D3 +/−), DYS single heterozygous (DYS +/−), and double D3 and DYS heterozygous (D3×DYS +/−) mice. Data are represented as means ± SEM and were analyzed with One−way ANOVA followed by Tukey’s HSD (* *p* < 0.05, ** *p* < 0.01, *** *p* < 0.001, **** *p* < 0.0001).

Finally, the mRNA levels of IL1β in the PFC of mice bearing the D3 hypofunction (F 3,16 = 7.21, *p* = 0.003—Figure 4A), TNFα (F 3,16 = 7.55, *p* = 0.002—Figure 4B), and IL6 (F 3,16 = 8.47, *p* = 0.0013—Figure 4C) were significantly higher than those of the other genotypes (IL1β: D3 +/− vs. WT: *p* = 0.007; D3 +/− vs. DYS +/−: *p* = 0.002; D3 +/− vs. D3×DYS +/−: *p* < 0.0001; TNFα: D3 +/− vs. WT: *p* = 0.012; D3 +/− vs. DYS +/−: *p* = 0.026; D3 +/− vs. D3×DYS +/−: *p* = 0.002; IL6: D3 +/− vs. WT: *p* = 0.003; D3 +/− vs. DYS +/−: *p* = 0.011; D3 +/− vs. D3×DYS +/−: *p* = 0.003, Tuckey’s post hoc).

Similar results were obtained in the striatum, where single mutant−induced D3 hypofunction resulted in significant differences in the mRNA levels of IL1β (F 3,16 = 10.61, *p* = 0.004), TNFα (PFC: F 3,16 = 13.72, *p* = 0.001), and IL6 (F 3,16 = 7.04, *p* = 0.003). In particular, Tuckey’s post hoc revealed significantly higher mRNA levels of the main proinflammatory cytokines in D3 +/− mice compared to the other genotypes (IL1β: D3 +/− vs. WT: *p* = 0.003; D3 +/− vs. DYS +/−: *p* = 0.0016; D3 +/− vs. D3×DYS +/−: *p* < 0.0004; TNFα: D3 +/− vs. WT: *p* = 0.0003; D3 +/− vs. DYS +/−: *p* = 0.011; D3 +/− vs. D3×DYS +/−: *p* = 0.0002; IL6: D3 +/− vs. WT: *p* = 0.037; D3 +/− vs. DYS +/−: *p* = 0.004; D3 +/− vs. D3×DYS +/−: *p* = 0.007).

In the HIPP, significant differences were found only for IL6 mRNA levels, which were significantly higher in D3 +/− mice compared to all the other genotypes (F 3,16 = 7.08, *p* = 0.002; D3 +/− vs. WT: *p* = 0.0008; D3 +/− vs. DYS +/−: *p* = 0.02; D3 +/− vs. D3×DYS +/−: *p* = 0.03, Tuckey’s post hoc) (Figure 4). No significant differences were found in the hippocampal expression levels of IL1β (F 3,16 = 1.6, *p* = 0.22) and TNFα among the genotypes (F 3,16 = 1.9, *p* = 0.16).

## 3. Discussion

Broad evidence indicates that schizophrenia is a complex and heterogeneous neurodevelopmental disorder reflecting an interplay of genetics and the environment [46]. Its complexity and heterogeneity are reflected by a lack of significant progress in the pharmacotherapy of this disabling disease.

To date, many susceptibility genes have been identified to be associated with schizophrenia. One of these is the DTNBP1 gene, which encodes DYS, whose genetic isoforms may alter cognitive responses to antipsychotic drugs through D2–mediated mechanisms. On the other hand, both in patients with schizophrenia and genetically modified mice, the concomitant reduction in D3 and DYS functionality is associated with improved executive and working memory abilities [32].

In this context, the use of mutant mice bearing selective heterozygosis for D3 and/or DYS—previously adopted by Leggio et al., 2021 [32]—allowed us to distinguish the transcriptional mechanism regulated by epistasis (gene–by–gene interaction) from those regulated by D3 and DYS independently.

In this study, we first replicated the experiments performed by Leggio and colleagues (2021) and investigated the differences in D3 and DYS mRNA levels among the genotypes [32] in the PFC and striatum. Additionally, in this study, we also included another area, the hippocampus, as a convergent body of research shows that structural and functional abnormalities of this area are involved in the pathophysiology of schizophrenia [32]. Supporting the strong connection between DYS function and D2–like receptors, D3 mRNA levels were significantly increased in all the areas when DYS +/− mice were compared to WT animals. Dys expression levels, in fact, are strictly linked to D2−like receptor recycling and trafficking [24,33]. Conversely, single mutant–induced D3 hypofunctioning resulted in a significant upregulation of DYS only in the STR.

Consistently with Leggio et al., (2021), here we demonstrated that the D3/DYS genetic interaction restored to the WT levels both the D3 mRNA levels in the PFC, HIPP, and striatum and striatal DYS expression observed, respectively, in their DYS and D3 single heterozygous counterparts. Given that reduced expression levels of DYS mRNA and protein have been found in the brain of patients with schizophrenia, our data suggest that the D3/DYS epistatic normalization of striatal DYS levels may ameliorate the pathophysiological alterations triggered by DYS reduction [24].

These regional differences (i.e., striatum versus PFC and HIPP) require further investigation as they may have important consequences in the clinical setting. In fact, genetic variations that result in decreased DYS functioning might predict critical differences in the efficacy and dosing of several D2–related antipsychotic drugs [15,47,48,49].

To better understand the molecular sequelae sustained by the epistatic interaction between D3 and DYS, we investigated the transcriptional effects mediated by DYS, D3, and their genetic interaction on the expression levels of key genes involved in neuroplasticity (i.e., GRIN and BDNF) and neuroinflammation (i.e., IL1β, TNFα, and IL6, [50] in the PFC, striatum, and HIPP, which are key brain areas implicated in schizophrenia [51].

Furthermore, alterations in the DYS complex have proven to contribute to synaptic and circuit deficits in key areas for schizophrenia, leading to alterations in the expression levels of post–synaptic neurotransmitter receptors, including the NDMA glutamate receptors [26].

Glutamate, the major excitatory neurotransmitter of the central nervous system, has been implicated in the pathogenesis of schizophrenia since the finding that NMDA receptor antagonists—including phencyclidine, ketamine, and CGS–19755 induced psychotic symptoms and impaired cognitive functions [52]. NMDA receptors are heterotetramers composed of two GRIN1 obligatory subunits and two regulatory subunits [53]. GRIN2A and GRIN2B are the main NMDAR regulatory subunits expressed in the limbic system; their expression undergoes a tight regulation in early postnatal development called developmental switch [53,54,55,56,57]. The progressive decrease in GRIN2B expression, which is high in embryonic life and lower in adults, is coupled with an opposite trend for GRIN2A, expressed at its lowest during the embryonic period and reaching a plateau during adult life [58]. These physiological changes result in a gradual increase in the GRIN2A/GRIN2B ratio during development [58].

In this study, we found that in both the PFC and the hippocampus—but not the striatum—GRIN1 and GRIN2A mRNA levels were downregulated in the context of reduced DYS or D3 expression. These data are consistent with those of Karlsgodt et al. (2010), who showed that decreased DYS levels are associated with specific decreases in NMDA–evoked currents and GRIN1 expression in prefrontal pyramidal neurons [51]. The reduction in GRIN1 and GRIN2A in mice bearing DYS and D3 single heterozygosis is consistent with the glutamate hypothesis for schizophrenia which specifies a role for NMDA receptors in either causing glutamatergic dysfunction or mediating cognitive and behavioral sequelae [59]. Determination of protein levels and their subcellular localization in these mice will help to elucidate whether the observed decrease in GRIN1 levels may be considered a direct measurement of the NMDAR amount [53]. Interestingly, the D3/DYS epistatic interaction reverted the GRIN1 and GRIN2A mRNA levels to those of WT mice.

Furthermore, the reduced expression of GRIN2A, caused by either D3 or DYS hypofunctioning, was not associated with changes in GRIN2B mRNA levels. This observation, together with other evidence both in vitro and in vivo [60,61], suggests that GRIN2A and GRIN2B expressions may be regulated independently of each other, and no compensatory expression of GRIN2B was induced in both D3 +/− and DYS +/− animals. It is possible that the reduction in GRIN2A expression and the lack of change in GRIN2B levels led to a decrease in the GRIN2A/GRIN2B ratio. Thus, as our data suggest that D3 and DYS hypofunction might affect the glutamate system in the PFC and HIPP differently compared with the striatum, further studies will be needed to understand if the effect of the D3/DYS interaction may affect NMDA trafficking as well and may impact differently on these regions due to their differing connectivity and developmental trajectories.

Impaired hypofunction of the dopamine system and NMDA–dependent glutamate transmission may sustain the cognitive deficits of schizophrenia by affecting intracellular trafficking and signaling of BDNF [62].

Lower levels of this neurotrophin have been associated with poorer cognitive functions in chronic patients, first episodes, and at−risk mental states [29]. Interestingly, while BDNF mRNA levels were not affected in single D3 or DYS mutant mice, DYSxD3 +/− mice have higher BDNF mRNA levels in all the areas investigated. Given the key role played by BDNF in promoting neuronal development, survival, and synaptic plasticity, our molecular data further corroborate the meaning of the D3−DYS genetic interaction in improving memory performances [34,63].

Although there is a large number of genetic and environmental factors conferring increased risk for schizophrenia, growing evidence indicates that neuroinflammation plays a pivotal role in the cascade of events leading to psychiatric disorders and neurodegenerative diseases [64].

In particular, during the last decade, dopamine has been shown to be a major regulator of inflammation [65]. Here, we demonstrated that hypofunctioning of D3–signalling resulted in higher mRNA levels of the main pro–inflammatory cytokines regulated by NF–kB (i.e., IL6, TNFα, and IL1β).

Dopamine D3 receptor expression and function are known to be downregulated by stressful stimuli, which are often associated with increased pro–inflammatory cytokine expression within the central nervous system [32]. Moreover, the downregulation of D3 in the nucleus accumbens was associated with a shift toward a proinflammatory response of microglia both in vitro and in vivo [43,66]. D3 genetic hypofunction was associated with increased mRNA levels of IL1β, TNFα, and IL6, mainly in PFC and the striatum, but in the context of reduced DYS, the increased pro–inflammatory cytokine expression observed in D3 +/− mice were normalized to WT levels. It is possible that the D3/DYS epistatic interaction may ameliorate schizophrenia–related phenotypes by mediating the crosstalk between the dopaminergic and immune systems.

To our knowledge, this is the first—albeit preliminary—study showing that the epistatic interaction between the dopamine D3 receptor and dysbindin–1 differently modulates the expression levels of key genes involved in neuroplasticity and neuroinflammation in the hippocampus, striatum, and prefrontal cortex, which are key brain areas implicated in schizophrenia.

As a preliminary study, this research has some limitations, which we would like to address in future studies.

First, we plan to test mice bearing selective heterozygosis for D3 and/or DYS for schizophrenia–like behaviors using several behavioral tasks for positive, negative, and cognitive symptoms [67]. Second, the animal models used in this study had proven to be valid models for uncovering the complex mechanisms involved in the DYS/D3 interaction, encouraging the study of this interaction in a larger sample and investigating gender differences [68]. Third, the gene expression analyses performed in this study suggest a role for DYS and D3 in modulating neuronal signaling altered in schizophrenia. Although differences in mRNA levels do not necessarily reflect differences in protein levels [69], our data encouraged us to perform proteomic and metabolomic studies in the near future.

Finally, the evidence gathered so far suggests that DYS and D3 hypofunctioning and their functional interaction exert area−dependent effects. We plan to perform additional experiments in other brain areas for schizophrenia, including the amygdala [70], hypothalamus [71], and midbrain [72], as well as to distinguish between the ventral and dorsal hippocampus [73]. Not least, as the results suggested—for the first time—that DYS and D3 hypofunctioning and their functional interaction induce different transcriptional effects on the expression levels of key proinflammatory cytokines, we plan to include in future studies peripherical areas such as the thymus and spleen, given their key role in immune response [74].

In conclusion, this study provides new insights into the impact of the D3/DYS interaction in regulating mRNA levels of neuroplasticity and inflammatory–related genes in three key brain areas for schizophrenia. Our results pave the way to future studies which may help to clarify the genetic mechanisms and functional interactions involved in the etiology and development of this psychiatric disorder.

## 4. Materials and Methods

### 4.1. Animal Models

The mouse line was generated by breeding D3 −/− mice with DYS −/− mice to obtain double D3 and DYS heterozygous (D3×DYS +/−) mice as previously described [32]. Both lines were on a C57BL/6J genetic background.

A breeding scheme consisting of mating one D3 +/− × DYS +/− male mouse with two C57BL6/J female mice was followed [29]. Littermates were screened and 3− to 6−month−old male wild−type (WT—D3×DYS +/+), D3 single heterozygous (D3 +/−), DYS single heterozygous (DYS +/−), and double D3 and DYS heterozygous (D3×DYS +/−) mice were employed in this experiment as in the previous studies [32].

Animals were checked daily for signs of discomfort as indicated by the animal care and use guidelines (National Academy of Sciences. Guide for the Care and Use of Laboratory Animals, 1998, “Guidelines for the Care and Use of Mammals in Neuroscience and Behavioral Research” (National Research Council 2003)). All procedures were carried out in accordance with the EC guidelines (EEC Council Directive 86/609 1987) and the Italian legislation on animal experimentation (Decreto Legislativo 26/2014) and had the approval of the local Ethical Committee.

### 4.2. Brain Areas Collection

Brains were harvested, immediately snap−frozen in liquid nitrogen, and then stored at −80 °C for further analysis. The prefrontal cortex (3.20 to 2.46 mm bregma), the striatum, and the hippocampus of each animal (N = 5 for each genotype) were dissected and used for RNA extraction. Tissues were collected from both hemispheres.

### 4.3. Total RNA Extraction, Reverse Transcription, and Real–Time Polymerase Chain Reaction

RNA extraction and DNAse treatment were performed using GenElute™ Mammalian Total RNA Miniprep Kit and DNase70–On–Column DNAse I Digestion Set (Sigma−Aldrich^®^, Milan, Italy—as previously described [32]). Two µg of total RNA was reverse transcribed with a High–Capacity cDNA Reverse Transcription Kit (Life Technologies Corporation, Carlsbad, CA, USA) in 20 µL of the reaction mix. For gene expression analysis, we used specific forward and reverse primers at the final concentration of 300 nM (Table 1), with 5 µL of cDNA, 10 µL of Bio–Rad SsoAdvanced Universal SyBR Mix, and topped to 20 µL with ddH_2_O. Real–Time PCR was performed in a Bio–Rad (Hercules, CA, USA) CFX Connect thermocycler running a custom program, which consisted of 95 °C for 30 s, 40 cycles of 95 °C for 15 s, and 60 °C for 30 s.

### 4.4. Statistical Analysis

Stability values were calculated for each candidate housekeeping gene cyclophilin A (CypA) and the ribosomal protein L27 (RPL27) using NormFinder (https://moma.dk/normfinder–software, accessed on 1 February 2023), taking into account intra- and intergenotype variations. The arithmetic mean of Cts of the reference genes was used as a calibrator. For an appropriate application of the comparative ΔΔCt method, we demonstrated that the amplification efficiency of the target genes and endogenous control genes were approximately equal.

The mRNA levels of D3 receptors, DYS, ionotropic glutamate receptors NMDA (GRIN1, GRIN2A, and GRIN2B), BDNF, and the pro–inflammatory cytokines IL6, IL1β, and TNFα were analyzed in the prefrontal cortex (PFC), striatum (STR), and hippocampus (HIPP) of wild−type (WT—D3×DYS +/+), D3 single heterozygous (D3 +/−), DYS single heterozygous (DYS +/−), and double D3 and DYS heterozygous (D3×DYS +/−) male adult mice (N = 5 for each genotype).

Data from single areas were analyzed with a One−way analysis of variance (ANOVA) followed by Tukey’s HDS post hoc test. Analyses were conducted using SPSS for Windows v.28 (SPSS Inc., Chicago, IL, USA).

Extreme outliers were excluded before statistical analysis using the boxplot tool in SPSS (more than 3× the interquartile range outside of the end of the interquartile box).

Figures were created using GraphPad Prism v8 (GraphPad Software, La Jolla, CA, USA).

## 5. Conclusions

This pivotal study shows interesting molecular mechanisms mediated by DYS, D3 receptors, and their functional interaction on the expression levels of key genes for neuroplasticity and neuroinflammation. In summary, the results of the current study help to clarify the interplay between DYS, D3, glutamate transmission, and inflammation, and their relevance in the pathogenesis of schizophrenia.

The evidence gathered so far suggests that DYS and D3 hypofunctioning and their functional interaction exert area–dependent effects. The animal models used in this study have proven to be valid models for uncovering the complex mechanisms involved in the DYS/D3 interaction. The molecular evidence for the role of DYS and D3 in modulating neuronal signaling altered in schizophrenia encouraged us to perform proteomic and metabolomic studies in the near future.

## Figures and Tables

**Table 1:** Primer sequences.

Gene Bank Accession	Target	Type Sequence
NM_008907.2	CypA	FW: AGCATACAGGTCCTGGCATC
		RV: AGCATACAGGTCCTGGCATC
NM_011289.3	RPL27	FW: AAGCCGTCATCGTGAAGAACA
		RV: CTTGATCTTGGATCGCTTGGC
NM_007877.2	D3	FW: GGCAGCCAACAGACAATGAA
		RV: GACTCGGAACTCCTTAAGCCC
NM_025772.4	DYS	FW: TGAAGGAGCGGCAGAAGTT
		RV: GTCCACATTCACTTCCATG
NM_008169	GRIN1	FW: GGCCTCCAGCTTCAAGAGAC
		RV: TCCCTATGACGGGAACACAG
NM_008170	GRIN2A	FW: CGCTACACACTCTGCACCAA
		RV: CCATTCCCGGTCCTTATTCA
NM_008171	GRIN2B	FW: CCACGAGAAGAGGATCTACC
		RV: CAGAAGGATTATCACCAGCTT
NM_007540.4	BDNF	FW: CCATAAGGACGCGGACTTGTAC
		RV: AGACATGTTTGCGGCATCCAGG
NM_008361	IL1β	FW: TGAAAGCTCTCCACCTCAATG
		RV: CCAAGGCCACAGGTATTTTG
NM_013693.2	TNFα	FW: GGCCTCCCTCTCATCAGT TC
		RV: CACTTGGTGGTTTGCTACGA
NM_031168	IL6	FW: CTTCACAAGTCGGAGGCT TA
		RV: CAAGTGCATCATCGTTGT TC

## Data Availability

The data that support the findings of this study will be made available on request.
